# Morphological and Molecular Profiling of Amyloid-β Species in Alzheimer’s Pathogenesis

**DOI:** 10.1007/s12035-024-04543-4

**Published:** 2024-10-24

**Authors:** Zaida L. Almeida, Daniela C. Vaz, Rui M. M. Brito

**Affiliations:** 1 Chemistry Department and Coimbra Chemistry Centre - Institute of Molecular Sciences (CQC-IMS), University of Coimbra, 3004-535 Coimbra, Portugal; 2 School of Health Sciences, Polytechnic Institute of Leiria, 2411-901 Leiria, Portugal; 3 LSRE-LCM, Laboratory of Separation and Reaction Engineering and Laboratory of Catalysis and Materials, Leiria, 2411-901 Portugal; 4 ALiCE - Associate Laboratory in Chemical Engineering, University of Porto, 4200-465 Porto, Portugal

**Keywords:** Alzheimer’s disease (AD), Amyloid-beta (Aβ) peptide, Tau protein, Protein aggregation, Amyloid plaques, Aβ-based therapies

## Abstract

Alzheimer’s disease (AD) is the most common form of dementia around the world (~ 65%). Here, we portray the neuropathology of AD, biomarkers, and classification of amyloid plaques (diffuse, non-cored, dense core, compact). Tau pathology and its involvement with Aβ plaques and cell death are discussed. Amyloid cascade hypotheses, aggregation mechanisms, and molecular species formed in vitro and in vivo (on- and off-pathways) are described. Aβ42/Aβ40 monomers, dimers, trimers, Aβ‐derived diffusible ligands, globulomers, dodecamers, amylospheroids, amorphous aggregates, protofibrils, fibrils, and plaques are characterized (structure, size, morphology, solubility, toxicity, mechanistic steps). An update on AD-approved drugs by regulatory agencies, along with new Aβ-based therapies, is presented. Beyond prescribing Aβ plaque disruptors, cholinergic agonists, or NMDA receptor antagonists, other therapeutic strategies (RNAi, glutaminyl cyclase inhibitors, monoclonal antibodies, secretase modulators, Aβ aggregation inhibitors, and anti-amyloid vaccines) are already under clinical trials. New drug discovery approaches based on “designed multiple ligands”, “hybrid molecules”, or “multitarget-directed ligands” are also being put forward and may contribute to tackling this highly debilitating and fatal form of human dementia.

## Alzheimer’s Disease

Alzheimer’s disease (AD) is the most common form of dementia with 60–70% of cases according to the World Health Organization (WHO) (who.int). AD is a chronic and progressive multifactorial neurodegenerative disease affecting the central nervous system (CNS). This pathology is defined by the simultaneous presence of different filamentous amyloid inclusions in the brain, such as abundant extracellular deposits and neuritic plaques (NPs) of amyloid-beta (Aβ) and intraneuronal neurofibrillary tangles (NFTs) of hyperphosphorylated Tau protein. AD leads to progressive dysfunction and death of neurons, resulting in a gradual loss of cognitive abilities and memory, as well as personality changes, thoughts, and behavior, leading to the patient’s death from complete brain failure [1]. After diagnosis, AD patients have an average survival period of 7–10 years [2]. In 2019, according to the WHO, AD was the seventh-leading cause of death worldwide, the fourth-leading cause of death globally among the elderly population above the age of 70 [3], and one of the major causes of disability and dependency among older people. In the last 30 years, there has been a significant increase in the mortality rates of dementia worldwide. The death rate has almost doubled, from 1990 to 2019, rising from 10.49 deaths per 100,000 to 20.98 deaths per 100,000 [4]. The disease progresses symptomatically from mild to severe with a higher prevalence in older people, namely 5% of people aged 65 to 74, 13% of people aged 75 to 84, and 33% of people aged 85 and older [5].

**Fig. 1:**
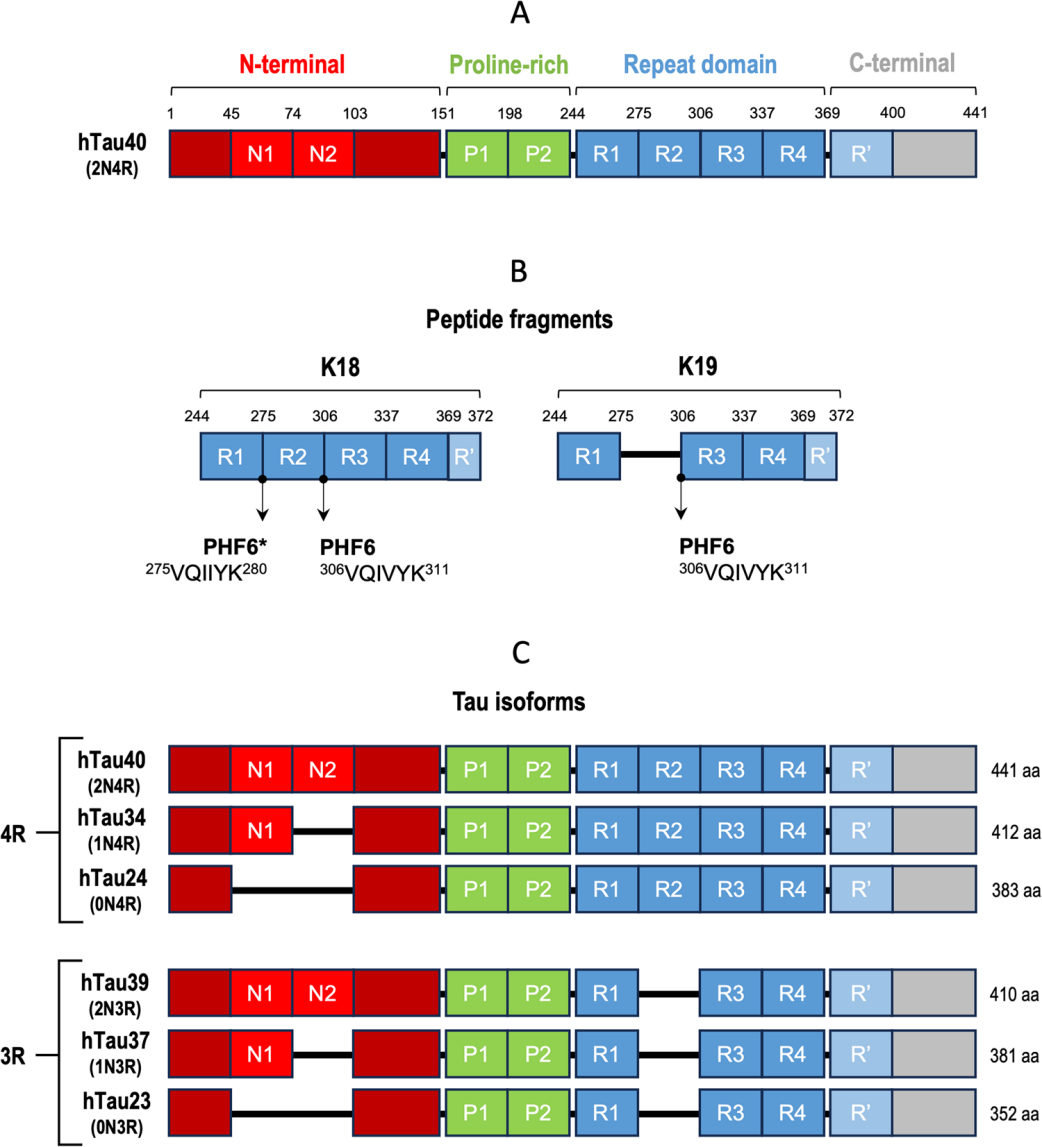
Domains and isoforms of the Tau protein. **A** Tau consists of 4 primary domains/regions: the N-terminal domain (red), the proline-rich domain (green), the repeat domain (RD) or microtubule-binding domain (blue), and the C-terminal region (gray). The CNS isoform hTau40, which is the longest one, consists of 441 amino acids and includes regions N1 and N2, as well as R1, R2, R3, and R4 (2N4R). RD is responsible for the formation of Tau filaments, working as a structural backbone. **B** Models for the formation of β-sheet-structures often employ Tau-based peptide fragments. The K18 fragment presents the R1-R2-R3-R4 domain, while the K19 fragment presents the R1-R3-R4 domain. Assembly of β-sheets is facilitated by the hexapeptides PHF6 and PHF6*. **C** Tau isoforms with lengths of 352 to 441 amino acids. Alternative splicing of the sub-domains N1, N2, and R2 results in the production of six isoforms within the CNS. The repeat domains R1, R3, and R4 are consistently present, while R2 is exclusively included in the three 4R isoforms. Skipping of N1 and/or N2 can occur, though the inclusion of N2 necessitates the inclusion of N1 as well. Consequently, the resultant variants encompass 0N3R (hTau23), 1N3R (hTau37), 2N3R (hTau39), 0N4R (hTau24), 1N4R (hTau34), and 2N4R (hTau40) Tau isoforms. Adapted from references [12, 13]

There are two forms of AD. One is early-onset AD, also known as familial AD (FAD), which is an unusual form of the disease seen in nearly 5.5% of the cases and occurs before the age of 65 [6]. This form of AD results from mutations in three major genes: the amyloid precursor protein (APP) gene, the presenilin 1 (PSEN1) gene, and the presenilin 2 (PSEN2) gene, leading to an increase in β- and γ-secretase cleavage activity, respectively (Fig. 1). Mutations in these genes induce the abnormal overproduction of Aβ [7]. The other form of AD is late-onset AD, termed sporadic AD (SAD), which is the more common form of the disease affecting anyone at any age but, usually, occurs in those above the age of 65. The cause of SAD is still not well understood. However, it is believed to be determined by a combination of genetic, environmental, and lifestyle factors [7]. Age is the main risk factor [8], but individuals may also present a genetic predisposition in 60 to 80% of the cases [9]. However, other risk factors have been identified, such as gender (women have a two-fold increased risk of AD compared to men), traumatic brain injury, heart disease, stroke, hypertension, obesity, type 2 diabetes, high cholesterol levels, lack of vitamin D, low levels of antioxidants, diets rich in saturated and trans fatty acids, depression, stress, environmental pollution, physical inactivity, social isolation, marital status, low academic level, smoking, alcohol in excess, inadequate sleep, and metabolic syndrome [10, 11]. In addition, older African American and Hispanic/Latino adults are more likely to develop AD than older white adults (fda.gov).

Currently, approximately 55 million people worldwide are living with AD or other forms of dementia. Alzheimer’s Disease International (ADI) (alz.co.uk) estimates this number will almost double every 20 years, reaching 78 million in 2030 and 139 million in 2050. In conformity with the WHO, due to the growing aging population, AD has become a major public health concern, with global costs at around 1.3 trillion US dollars in 2019.

Clinical and post-mortem neuropathological progression of SAD involves the accumulation of amyloid plaques and neurofibrillary tangles. This situation led to AD being clinically redefined by the National Institute on Aging–Alzheimer’s Association (NIA-AA) [14]. There are three pillars underlying this approach: (a) the neuropathological evidence of AD, (b) biochemical and neuroimaging biomarkers, and (c) clinical symptoms.

### Neuropathology

NIA-AA guidelines consider amyloid plaques and neurofibrillary tangles essential neuropathologic features of AD [15, 16]. The main points to consider are (1) the recognition that AD may occur in the absence of cognitive impairment; (2) the consideration of an “ABC” score for AD neuropathological changes, incorporating histopathologic assessment of Aβ deposits (called A, based on Thal phases — see in more detail Sect. Localization and Morphology of Deposits and Neuritic Plaques of Amyloid-β in vivo), staging of NFTs (called B, based on Braak stages — see in more detail Sect. Tau Protein, Neurofibrillary Tangle Localization, and Tau Hypothesis), and scoring of neuritic plaques (called C, based on the Consortium to Establish a Registry for Alzheimer’s Disease (CERAD) — see in more detail Sect. Localization and Morphology of Deposits and Neuritic Plaques of Amyloid-β in vivo); and (3) the assessment of comorbidities, such as vascular brain injury which modifies the clinical presentation for each individual.

According to the Aβ cascade hypothesis, which is believed to be the origin and trigger of AD, the presence of Aβ plaques is considered an essential condition for the neuropathological diagnosis of SAD by the NIA-AA guidelines. On the other hand, the presence of NFTs alone is not considered a prime manifestation of SAD.

### Biomarkers

Cerebrospinal fluid (CSF), plasma, and blood biomarkers used in AD diagnosis are Aβ42, Aβ42/Aβ40 ratio, phosphorylated Tau or phospho-Tau (P-Tau), total Tau (t-Tau), P-Tau ratio, neurofilaments, synaptic proteins, activated astrocytes, and inflammatory markers [17]. The available methods cannot detect small levels of Tau, P-Tau, Aβ, and structural or synaptic proteins until the degenerative process has progressed to at least the intermediate stages of AD (A2B2C2, according to the ABC score).

Computed tomography (CT) and magnetic resonance imaging (MRI) uncover hippocampal atrophy as a late indicator of AD, only manifested in the presence of escalated NFT pathology and neuronal diminution within the hippocampus. ^18^F-fluorodeoxyglucose positron emission tomography (^18^F-FDG PET) and functional MRI (fMRI) exhibit the potential to detect hypo-perfusion and hypo-metabolism associated with neuronal activity [18]. Amyloid-PET and Tau-PET utilize specific radiotracers that facilitate the visualization of aberrant protein aggregations, notably Aβ and P-Tau variants, correspondingly. Tau-PET detects initial Tau depositions in the entorhinal and temporal cortices among Aβ-negative non-demented subjects, with subsequent dissemination to other cerebral regions following advanced NFT Braak stages in subjects with concomitant Aβ pathology [18].

In 2018, the NIA-AA defined AD based on the AT(N) biomarker system, which assessed the presence of Aβ plaques (A), fibrillar Tau (T), and neurodegeneration or neuronal injury (N) to categorize the presence and extent of AD [14]. Among the biomarkers, detection of “A” includes cortical amyloid PET ligand binding and low CSF Aβ42 or low CSF Aβ42/Aβ40 ratio. Detection of “T” considers elevated CSF P-Tau and cortical Tau PET ligand-binding, while the biomarkers for “N” are CSF t-Tau, ^18^F-FDG PET hypometabolism, and medial temporal lobe atrophy on MRI. Individuals can have 8 possible biomarker combinations, namely A^−^T^−^N^−^, A^−^T^−^N^+^, A^−^T^+^N^+^, A^+^T^+^N^+^, A^+^T^+^N^−^, A^+^T^−^N^+^, A^+^T^−^N^−^, and A^−^T^+^N^−^ [14].

### Clinical Classification of AD

NIA-AA states that AD is a clinically progressive neurodegenerative disease and in terms of symptomatology can be categorized as pre-clinical AD (stages 1 and 2), mild cognitive impairment (MCI) due to AD (stage 3), and mild, moderate, and severe AD dementia (stages 4 to 6) [14, 19–23]. Individuals with pre-clinical AD exhibit measurable changes in AD biomarkers in the brain, even before experiencing major symptoms such as memory loss. Pre-clinical stage 1 refers to asymptomatic individuals with abnormal amyloid biomarkers, and pre-clinical stage 2 refers to individuals with subtle cognitive dysfunction, cognitive decline, and mild neurobehavioral symptoms. It is crucial to emphasize that the initial detection of positive Tau-PET within the inner temporal cortex, lacking concurrent positive Aβ biomarkers, does not warrant classification as pre-clinical AD under the current criteria established by NIA-AA, which fits with the principles of the amyloid cascade hypothesis. Stage 3 pertains to individuals exhibiting MCI alongside abnormal amyloid and injury biomarkers. Mild to severe AD dementia stages 4 to 6 refer to the gradual progression of levels of cognitive impairment in individuals and have an impact on the ability to perform basic activities of daily living, as well as loss of independence.

In the clinical context, some neuropsychological test batteries can be used to standardize procedures for the evaluation and diagnosis of patients with AD, namely the Consortium to Establish a Registry for Alzheimer’s Disease (CERAD) examination, the Mini-Mental State Examination (MMSE), and various other test constructs and scales, like the Clinical Dementia Rating (CDR) that investigates different aspects of memory over a broad range of various cognitive domains [24–26].

## Tau Protein, Neurofibrillary Tangle Localization, and Tau Hypothesis

Tau is a highly soluble and natively intrinsically disordered protein (IDP), mainly expressed in neurons, and involved in the stabilization and organization of microtubules in axons, which are essential for maintaining the integrity of neurons [27]. In the CNS, Tau protein exists in 6 isoforms having four major primary domains: the N- and C-terminal regions, the proline-rich domain, and the repeat domain (RD) or microtubule-binding domain. The isoforms exhibit discrepancies in their composition due to three alternatively spliced exons, leading to the generation of Tau isoforms with 0, 1, or 2 inserts within the N-terminal projection domain (N0, N1, and N2 isoforms), along with 3 or 4 pseudo-repeats (3R and 4R isoforms) in the Tau repeat domain (Tau RD) (see Fig. 1) [28]. Within the adult human brain, both 3R and 4R Tau isoforms are expressed, predominantly localized within the axons of neurons under typical physiological conditions.

**Fig. 2:**
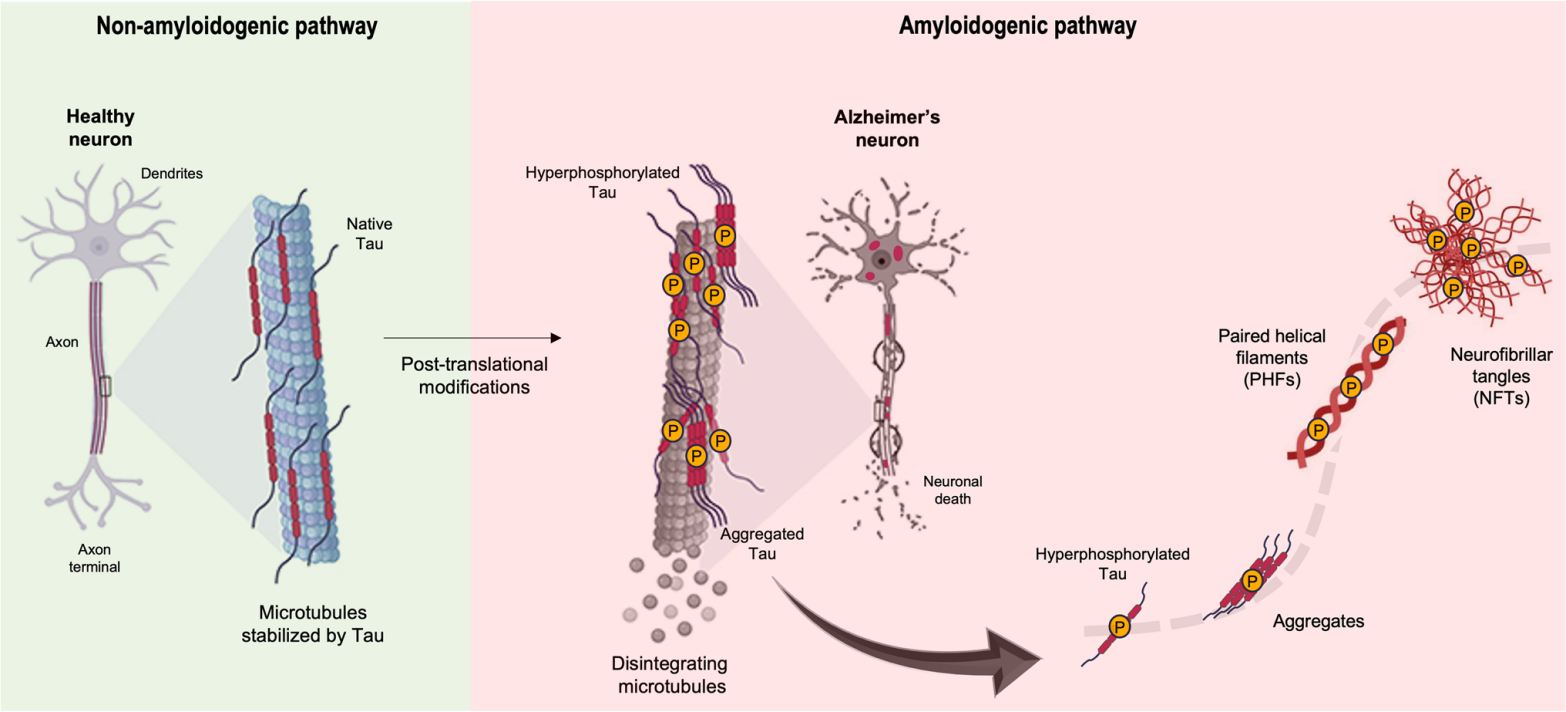
Schematic representation of the formation of neurofibrillary tangles (NFTs) by the Tau protein in Alzheimer’s disease. In a non-amyloidogenic pathway, functional Tau is believed to play a role in the stabilization of axonal microtubules in neurons. However, under pathological conditions (amyloidogenic pathway), Tau becomes hyperphosphorylated and disconnects from microtubules. Phosphorylated Tau then aggregates via a nucleation-dependent mechanism (sigmoidal dash line) forming paired helical filaments (PHFs) and then NFTs that further lead to neuronal death. Adapted from reference [32]

The physiological functions of Tau are regulated by a variety of post-translational modifications, e.g., phosphorylation, glycation, and acetylation, among others [29]. Although some authors claim that Tau phosphorylation occurs after aggregation [30], there is a significant body of evidence that hyperphosphorylation of Tau leads to its detachment from microtubules and pathological Tau aggregation in the CNS [31] (Fig. 2). The most extended Tau molecule found in the CNS denoted as hTau40 or 2N4R consists of 441 residues. Within its structure, there are potentially 85 sites available for phosphorylation, encompassing serine (Ser), threonine (Thr), or tyrosine (Tyr) residues, alongside 102 hydrophobic residues, including alanine (Ala), valine (Val), isoleucine (Iso), leucine (Leu), methionine (Met), and phenylalanine (Phe) residues. Hyperphosphorylated Tau has the ability to assemble into paired helical filaments (PHFs) inside neurons that evolve to form NFTs (Fig. 2). The aggregation of neurofibrillary tangles (NFTs) within neuronal intracellular compartments has the potential to disrupt the usual cytoskeletal organization, resulting in subsequent neuronal dysfunction and eventual cell death.

Tau aggregation is strongly driven by two hexapeptide fragments within Tau, PHF6* (^275^VQIINK^280^) and PHF6 (^306^VQIVYK^311^) (Figs. 1 and 2), both located in the microtubule-binding region. The PHF6 sequence is in the third repeat (R3) and is present in all Tau isoforms. In turn, the PHF6* sequence is in the second repeat (R2) and can only be found in four-repeat (4R) Tau isoforms. Hexapeptide motifs have the highest predicted potential for β-structure within the Tau sequence. Mutations in the hexapeptide regions can alter β-propensity, which may result in an increase or decrease in aggregation [33–35]. Tauopathies are categorized based on the predominant presence of Tau isoforms containing either 3R or 4R within the microtubule-binding domain. For instance, AD is characterized by the aggregation of Tau isoforms encompassing both 3R and 4R repeats. Conversely, corticobasal degeneration primarily exhibits aggregates composed of Tau 4R isoforms, while Pick’s disease predominantly features 3R aggregates [36, 37].

**Fig. 3:**
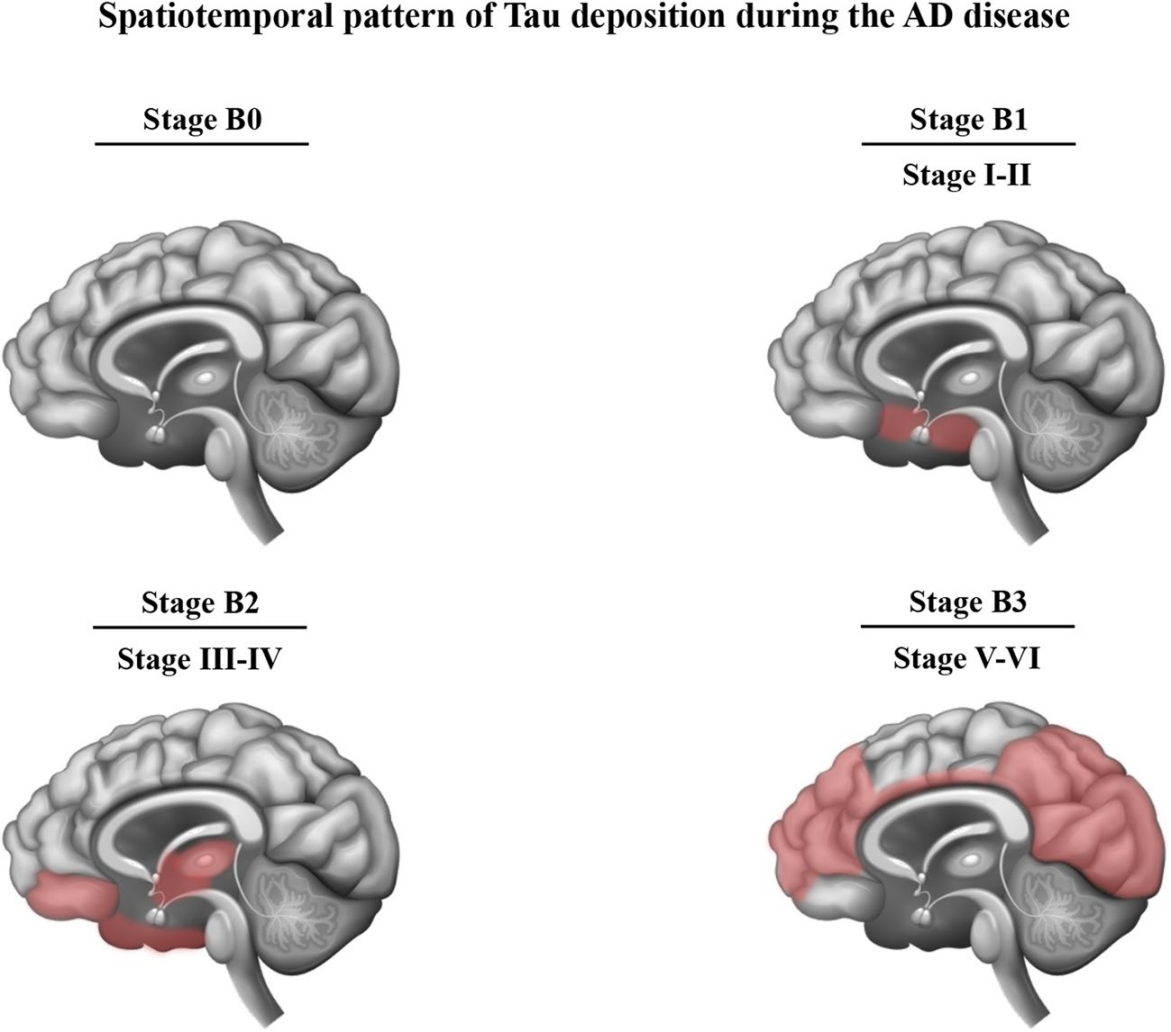
Spatiotemporal pattern of NFTs deposition during the AD disease cascade in the human brain according to Braak stages and “B” score. In Braak stages I–II, modifications primarily occur within the superficial layers of the transentorhinal cortex (referred to as transentorhinal stages). Braak stages III–IV exhibit extensive involvement of both the transentorhinal and entorhinal regions, with comparatively milder engagement of the hippocampus and various subcortical nuclei (designated as limbic stages). Braak stages V–VI show profound neurofibrillary pathology development within neocortical association areas (known as isocortical stages), along with a progressive escalation of pathology within brain regions affected during stages I–IV. Accordingly, Braak stages I–II, III–IV, and V–VI are scored as B1, B2, and B3, respectively. Stage B0 depicts no symptoms. Light red areas represent the regions affected by NFT deposition for each stage of neuropathology. Adapted from references [39, 44, 45]

In preclinical models, the pathological aggregation of Tau has been observed to adversely impact neuronal function [38]. Furthermore, this aggregation pattern follows a stereotypical spread across different brain regions, exhibiting a strong correlation with the severity of the disease [39] (Fig. 3). Tau pathology propagation, which refers to the intraneuronal transfer of Tau pathology, has been shown to occur in a prion-like manner [40, 41]. A spatiotemporal course based on the progression of abnormal Tau in the form of NFTs deposition has been classified into 6 stages according to Braak stages [39, 42] and also staged from 0 to 3 referent to the “B” score in the ABC system. Stage B0 signifies the absence of Tau detected through immunohistochemical staining. Subsequent B-scores follow the Braak staging protocol, starting with the transentorhinal cortex (Braak stage I) and progressing to involve the entorhinal cortex and hippocampus (Braak stage II). The deposition of Tau then extends to encompass the temporal neocortex, inclusive of the occipitotemporal and lingual gyrus (Braak stage III) before further expansion to involve the middle temporal gyrus (Braak stage IV). Eventually, Tau aggregates disseminate throughout the remaining cortex (Braak stages V–VI), with occasional involvement of the basal ganglia. Consequently, Braak stages I–II, stages III–IV, and stages V–VI are categorized as stages B1, B2, and B3, respectively [43].

Analyses of a large number of human brains across the lifespan show that Tau pathology in AD precedes by several decades the formation of Aβ plaques without cognitive impairment [46]. The observation that the in vivo expression of hTau40 results in significant cortical and hippocampal neuronal loss in the absence of Aβ peptide accumulation provides compelling evidence that Tau-induced neurodegeneration can manifest independently of Aβ accumulation [47, 48]. Tau pathology has been also correlated with progressive gray matter loss and cognitive impairment without concomitant Aβ deposition [49] and progressive cognitive decline in SAD [50]. Cognitive decline generally correlates better with NFT burden rather than Aβ plaques [51]. Moreover, the identification of a woman harboring a rare mutation, who remained dementia-free despite extensive Aβ deposition but minimal Tau pathology, underscores the emerging concept that aberrant Tau may represent a pivotal etiological factor in AD [52]. Various investigations have demonstrated that neurons lacking Tau are resistant to Aβ-induced neurotoxicity in vitro and that reducing endogenous Tau levels in AD mouse models can mitigate Aβ-induced synaptotoxicity and memory impairments [53, 54]. Furthermore, interbreeding mice carrying human Tau mutations with AD transgenic models has been shown to accelerate the formation of NFTs and neuronal demise [55, 56]. Collectively, these findings support the Tau hypothesis, positing that tauopathy follows a highly selective pattern and sequential progression in AD. Thus, although still under some controversy and serious debate regarding which, how, and when the main molecular trigger of AD is formed, the Tau hypothesis proposes that pathogenic Tau protein is the primary factor that drives neurodegeneration in AD.

## Amyloid-β Peptide

**Fig. 4:**
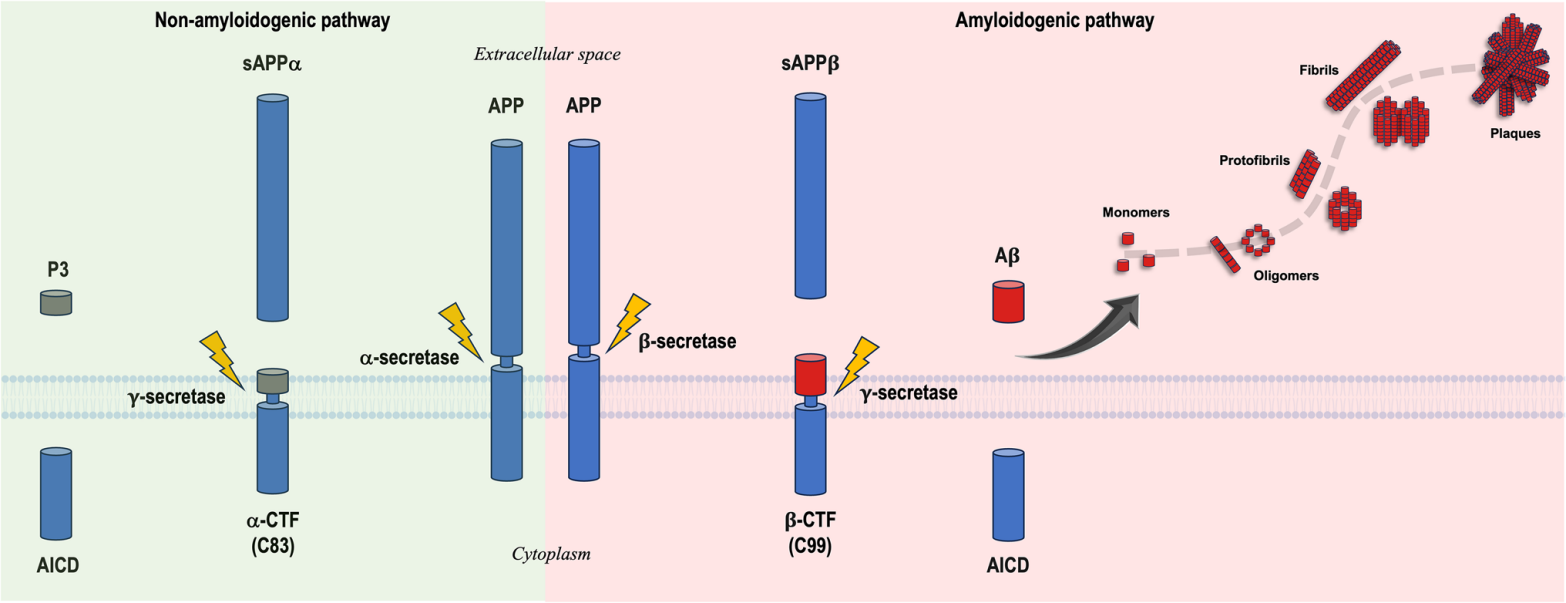
Amyloid precursor protein (APP) processing by secretase enzymes according to two different hydrolysis pathways. The non-amyloidogenic pathway shows a normal cleavage of APP by γ- and α-secretase, which leads to the release of the APP intracellular domain (AICD) and P3. The amyloidogenic pathway involves the cleavage of APP by the β-secretase enzyme to form β-CTF (or C99) and sAPPβ. Then, γ-secretase cleaves the resulting β-CTF, releasing the AICD and Aβ. Aβ monomers can assemble via a nucleation-dependent mechanism (sigmoidal dash line) to form higher-order structures, from oligomers to protofibrils, and eventually mature fibrils containing β-sheets which form the core component of amyloid plaques. Adapted from references [70, 71]

The amyloid precursor protein (APP) is a ubiquitous single-pass transmembrane protein that contains an extracellular domain, a hydrophobic transmembrane domain, and an intracellular domain in neurons [57]. Under physiological conditions, APP plays an essential role in neural growth and repair [58, 59]. APP can be cleaved by a combination of different secretase complexes, following two pathways. In the non-amyloidogenic pathway (Fig. 4), APP is predominantly cleaved by α-secretase, though the β-secretase pathway is part of normal physiology. In the amyloidogenic pathway (Fig. 4), the β-secretase pathway predominates. Cleavage by α- or β-secretase enzymes yields a protein fragment called secreted APP (sAPP) α or β, respectively. After a β-site APP cleavage by β-secretase or by the BACE enzyme, a 99 amino acid C-terminal fragment (CTF) is released. The β-CTF is then cleaved by γ-secretase, a multiprotease complex that includes proteins presenilin 1/2 (PSEN1/2), to release Aβ into the interstitial fluid (ISF) of the brain. The cleavage process performed by γ-secretase is not always the same, resulting in variations at the C-terminal end. These differences are responsible for the wide range of Aβ species that exist, which differ in size from 37 to 43 amino acids. The two primary components of the Aβ plaques of Alzheimer’s patients are Aβ40 and Aβ42, which are produced by cleavage at positions 40 and 42, respectively [60, 61]. Aβ40 is the most prevalent variant in vivo, comprising about 80% of the overall Aβ population. Minor amounts of the shorter Aβ38 and of the longer Aβ42 are produced (nearly 10% each), and very small amounts of Aβ37 and Aβ43 are generated (≤ 1%) [62]. However, Aβ42 is more amyloidogenic than Aβ40 given its tendency to aggregate due to the presence of two additional hydrophobic amino acids (isoleucine and alanine) at the C-terminus, and the Aβ42 soluble oligomers are considered to be more neurotoxic as well [63]. Deposition of Aβ40 is reported mainly in the cerebral vasculature [64], whereas Aβ42 is found predominantly in the parenchyma [65]. Genetic mutations associated with FAD (see Alzforum database alzforum.org/mutations) increase the concentration of Aβ [66], the Aβ42/Aβ40 ratio [67, 68], and/or the assembly of Aβ42 into amyloid plaques [69].

Amyloid-β peptides (Aβ40 and Aβ42) are intrinsically disordered proteins (IDP) with a molecular weight of approximately 4.3 kDa and 4.5 kDa and isoelectric points (pI) near 5.4 and 5.5, respectively. The concentration of Aβ in the CSF is in the picomolar range, namely 894 ± 759 pM for Aβ40 and 139 ± 202 pM for Aβ42 in control subjects, while for AD patients is 981 ± 409 pM for Aβ40 and 74 ± 42 pM for Aβ42 [72]. Aβ is found not just in the CNS, but also in the liver, kidneys, and muscles [73–75]. Research utilizing physiological concentrations of Aβ has indicated its involvement in regular synaptic function, facilitating long-term potentiation, supporting neuronal survival, and stimulating neurogenesis in neural progenitor cells [74–76]. Furthermore, Aβ has been implicated in the maintenance of the integrity of the blood–brain barrier (BBB), aiding in the repair of BBB disruptions, facilitating recovery from injuries, and exhibiting antimicrobial and tumor suppressor properties. [71, 76]. Moreover, Aβ is also known to bind transthyretin (TTR) [77], one of the most abundant proteins of the CSF [78].

### Amyloid Cascade Hypothesis for Alzheimer’s Disease: Pros and Cons

Several pathogenic conditions are believed to accelerate the progression of AD in the early stages of the disease. These factors cause significant destruction of brain areas. Various hypotheses have been put forward for the pathophysiology of this neurodegenerative amyloidosis, including the amyloid cascade hypothesis [79], the Tau hypothesis [80], the cholinergic hypothesis [81, 82], the glutamatergic or excitotoxic hypothesis [83], the oxidative stress hypothesis [84], the calcium signaling hypothesis [85, 86], the metal ion hypothesis [87], the apolipoprotein E (apoE) hypothesis [88], the GSK-3 hypothesis [89], the CREB signaling hypothesis [90], the vascular hypothesis [91], and the type 3 diabetes hypothesis [92].

The amyloid cascade hypothesis, proposed by Hardy and Higgins in 1992 [79], has been the dominant model of AD progression for over 30 years and states that Aβ aggregation initiates and drives AD pathogenesis. The formation and accumulation of Aβ plaques outside neurons appear to be the most significant pathological events in the development of AD several years before the onset of clinical signs and symptoms [93, 94]. This accumulation triggers a complex cascade of downstream events, such as activation of microglia and astrocytes, inflammatory responses, cytokine secretion, altered neuronal ionic homeostasis, altered kinase/phosphatase activities, mitochondrial dysfunction, oxidative stress, altered axonal transport, vascular damage, intracellular aggregation of hyperphosphorylated Tau protein, and synaptic dysfunction [95]. Collectively, these processes contribute to the gradual onset of neuritic damage and neuronal dysfunction resulting in cognitive decline, neuronal demise, and manifestation of dementia [95, 96].

This assumption is confirmed by multiple indications. Mutations in the three distinct genes (βAPP gene, presenilin 1 gene, and presenilin 2 gene) are highly linked to this hypothesis since they increase the amount of Aβ accumulated extracellularly [68, 97, 98]. Aβ levels begin to increase in the brains of people who are cognitively normal between the ages of 40 and 80 [99]. Various types of Aβ, ranging from soluble dimers to oligomers, whether synthetically produced or obtained from brains affected by AD, have demonstrated the capacity to induce synaptotoxic impacts and neuronal demise across a spectrum of in vitro and in vivo models and, therefore, seem to better correlate with AD symptoms and severity [100, 101]. The earliest symptoms of AD seem to be associated with Aβ plaques rather than Tau tangles [102]. In vitro cross-seeding between Aβ and Tau protein potentiates Tau aggregation [103, 104], and in animal models, injection of Aβ fibrils has been shown to induce Tau pathology [105]. Aβ formation in APP transgenic mice causes hyperphosphorylation of Tau, whereas there is no overt Aβ plaque pathology in Tau transgenic mice [106].

Despite the popularity of the amyloid hypothesis in AD research, this concept is not universally accepted due to contradictory evidence demonstrated in some cases over the years. Previous investigations exploring the efficacy of drugs targeting Aβ have indicated that reductions in amyloid plaques do not lead to alleviation of AD symptoms [107–111]. Recent findings from amyloid imaging studies have shown that elderly individuals without dementia exhibit comparable levels of Aβ plaques to those diagnosed with AD [107, 112–114]. In addition, some mouse models of AD have shown memory deficits before the development of Aβ plaques [115]. Some human neuropathological studies suggest that tangles may precede the formation of amyloid plaques [46]. All this together compelled a portion of the scientific community to reconsider this hypothesis.

### *Localization and Morphology of Deposits and Neuritic Plaques of Amyloid-β *In Vivo

**Fig. 5:**
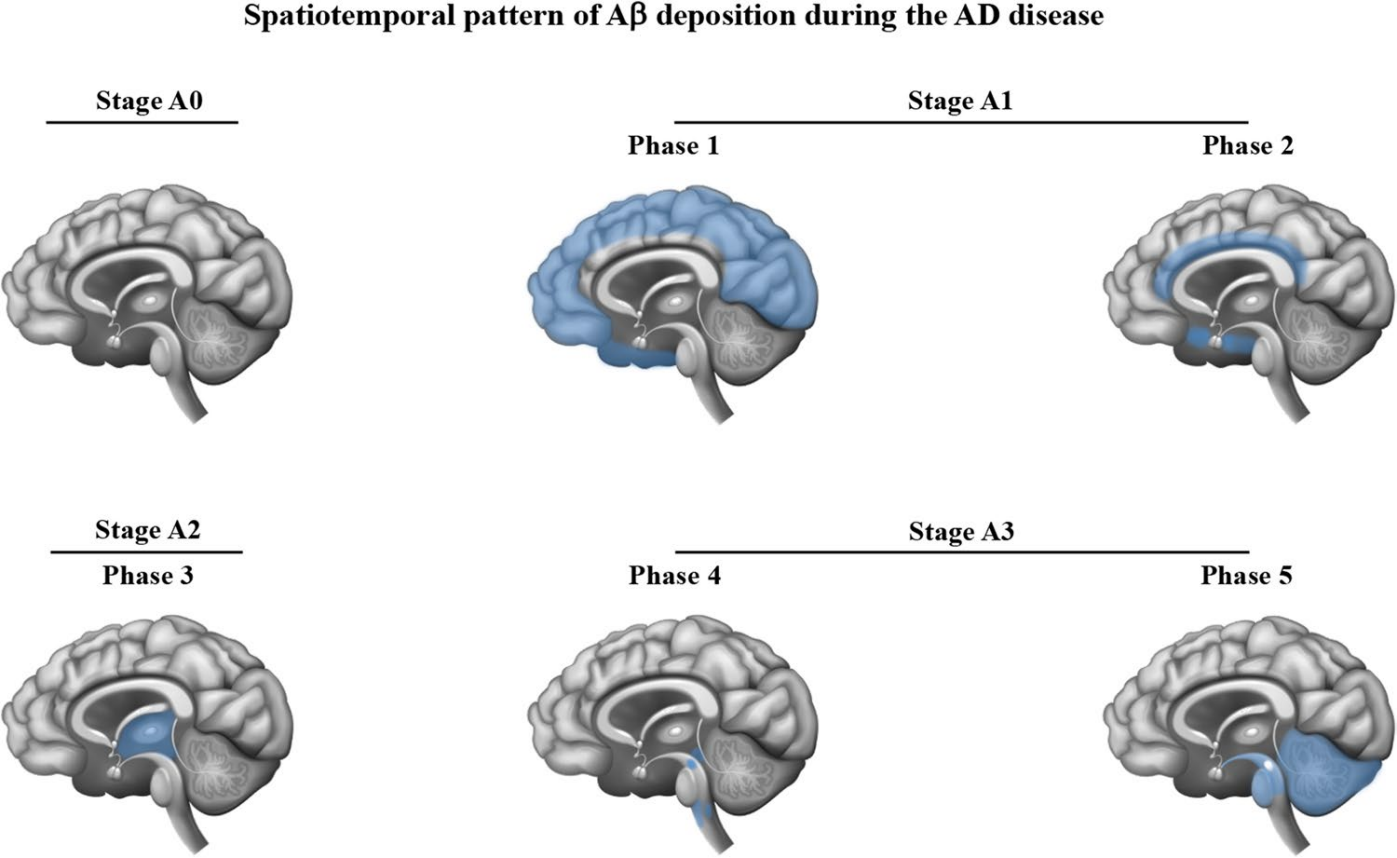
Spatiotemporal pattern of Aβ deposition during the AD disease cascade in the human brain according to the Thal phases and to the “A” score. Thal phase 1 delineates cortical regions exhibiting the initial buildup of Aβ during the early pre-clinical stage. Subsequent accumulation extends to allocortical regions and the midbrain in Thal phases 2 and 3, while Aβ deposition in the cerebellum and brainstem occurs during late-phase clinical stages (Thal phases 4 and 5). Stage A0 represents individuals with asymptomatic amyloidosis; stage A1 combines Thal phases 1 and 2; stage A2 is equivalent to Thal phase 3; and stage A3 combines Thal phases 4 and 5. Blue areas represent the regions affected by Aβ deposition for each stage of neuropathology. Adapted from references [44, 45, 116]

In the neuropathology analysis of the human brain in AD patients with different degrees of plaque accumulation, a spatiotemporal course of Aβ plaque formation has been classified into 5 phases, also coined as Thal phases, and staged from 0 to 3 relatively to the “A” score in the ABC system [45, 116–118] (Fig. 5).

The phases are based on a single parameter, the presence or absence of Aβ deposits in specific regions of the brain, without considering the quantity/density of Aβ present or the type of Aβ plaque. Stage A0 denotes an absence of amyloid in immunohistochemistry. Thal phase 1 is characterized by exclusively neocortical Aβ deposits. Aβ deposits are found in the frontal, temporal, parietal, and occipital cortices. Thal phase 2 demonstrates supplementary allocortical Aβ depositions in regions such as the entorhinal cortex, CA1, cingulate cortex, amygdala, presubiculum, and the fascia dentata. The combination of Thal phases 1 and 2 results in stage A1. In Thal phase 3, corresponding to stage A2, additional Aβ deposits emerge in diencephalic nuclei and in the striatum, encompassing structures such as the thalamus, hypothalamus, basal forebrain, caudate nucleus, putamen, claustrum, lateral habenular nucleus, and white matter. Thal phase 4 exhibits Aβ deposits in distinct brainstem nuclei including the substantia nigra, superior and inferior colliculi, inferior olivary nucleus, intermediate reticular zone, central gray of the midbrain, CA4, and the red nucleus. Finally, Thal phase 5 presents Aβ depositions in the cerebellum and additional brainstem nuclei such as the pons, locus coeruleus, reticular formation, raphe nuclei, parabrachial nuclei, and the dorsal tegmental nucleus. Thal phases 4 and 5 are consolidated into stage A3 of the ABC system.

**Fig. 6:**
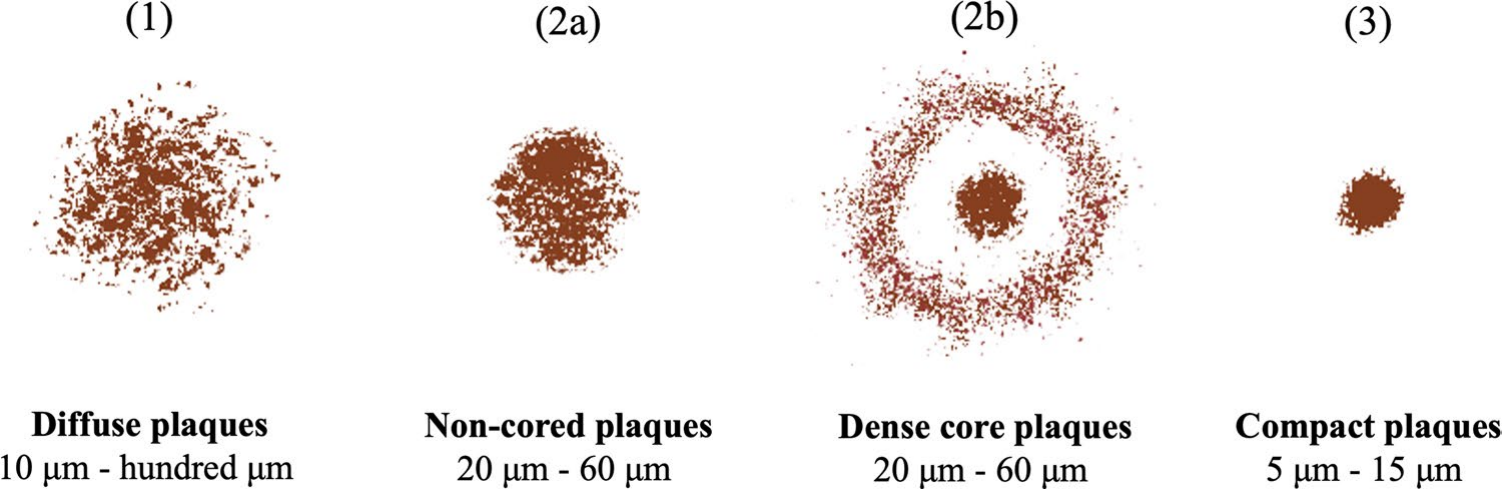
Illustration of different types of Aβ plaques found in AD patients. Adapted from reference [121]

The terminology for Aβ amyloid plaques can sometimes be confusing since several types of non-vascular amyloid deposits have already been described. Nevertheless, Aβ amyloid plaques and abnormal neurites are classified according to their morphology, distribution, and relative amounts. In this regard, there are three major types of Aβ inclusions with different morphological forms [119–121]:

1. Diffuse or pre-amyloid plaques (Fig. 6 (1)) predominantly consist of amorphous amyloid deposits with undefined boundaries, loosely arranged Aβ filaments, and a lack of dystrophic neurites [122, 123]. The size of these deposits can vary from 10 μm to several hundred μm. Notably, diffuse plaques do not elicit a glial response or lead to synaptic loss, so they are deemed insufficient for neuropathological diagnosis of AD. It is widely recognized that diffuse plaques represent the earliest form of plaque pathology in AD [124]. Furthermore, they are commonly observed in various brain regions of elderly individuals without cognitive decline, suggesting a lack of direct toxicity associated with these lesions [125, 126]. These plaques can be found in regions such as the entorhinal cortex, presubiculum, striatum, brainstem, cerebellum, and the subpial region of the isocortex. However, the duration for which diffuse deposits remain uncomplicated within the brain remains unknown. Aβ42 is the principal constituent of these plaques, which can be visualized with silver staining but exhibits weak staining for CR, ThS, and PIB (Pittsburgh compound B) [127–129].
2. Neuritic plaques (NPs)
3. Non-cored or primitive or immature neuritic plaques (Fig. 6 (2b)) are oval or spherical formations containing Aβ and altered neurites, with diameters ranging from 20 to 60 μm and lacking a densely packed Aβ region in the central portion [130]. These fibrillar plaques exhibit distinct pores and irregularities within their structure and are commonly observed in older individuals with AD [131]. Astrocytic and glial responses are frequently associated with these plaques, which can be stained with ThS [132].
4. Cored or classic or dense or mature or focal neuritic plaques (Fig. 6 (2b)) are compact cores ranging from 20 to 60 μm in diameter, encircled by loosely fibrillar deposits and predominantly containing Aβ42 [133, 134]. Adjacent to these plaques, there are Tau-positive dystrophic neurites, reactive astrocytes, and activated microglia [132, 135, 136]. Due to their association with neuronal loss and cognitive decline, these plaques serve as a hallmark for diagnosing AD. Dense core plaques are predominantly found in regions such as the hippocampus and the cerebral cortex, markedly increasing with age. In advanced AD cases, focal Aβ deposits become widespread. These plaques are detectable with silver staining and exhibit intense positivity with CR and ThS [135, 137, 138].
5. Compact or burnt-out plaques (Fig. 6 (3)) are 5–15 μm in diameter and characterized by a dense core lacking a surrounding neuritic component [139]. They test positive for ThS and are primarily constituted by Aβ40 [133].

A morphological progression has been proposed in which Aβ plaques originate as pre-amyloid diffuse deposits, evolve into primitive and/or mature NPs, and finally into burned-out plaques [120]. However, this progression and its development time course in human AD brains are still speculative.

The NPs density in neocortical areas can be scored according to the CERAD semi-quantitative scale and the “C” score of the ABC system, which can be categorized as none/diffuse, C0; sparse (1–5 NP/mm^2^), C1; moderate (6–19 NP/mm^2^), C2; and frequent (≥ 20 NP/mm^2^), C3, respectively [43, 140]. Diffuse plaques, which may be the initial morphological type of Aβ as stated before, can account for over 50% of plaque burden in preclinical cases but are not included in the CERAD classification system [132].

Aβ peptides not only deposit as amyloid plaques in the brain parenchyma, but also in the walls of blood vessels resulting in cerebral amyloid angiopathy (CAA) which may cause impaired blood flow, ischemic lesions, small infarcts, lobar intracerebral hemorrhages, and microbleeds. Amyloid deposits in CAA have a high Aβ40 and a low Aβ42 content and can affect small arteries, arterioles, and even capillaries of the gray matter of the cerebral cortices and of leptomeningeal vessels. A case was deemed to exhibit cerebral amyloid angiopathy (CAA) positivity when it demonstrated Aβ deposition in at least one leptomeningeal or cortical vessel, enabling reliable classification of CAA severity utilizing the Vonsattel grading system [141, 142]. This classification system categorizes CAA severity as either mild (Vonsattel grade 1), moderate, or severe (Vonsattel grades 2–4).

### *Comparison Between Amyloid-β Aggregation Mechanisms and Species Formed *In Vitro* and *In Vivo

The etiology of AD remains a central question for the scientific field, but the current thinking is still largely dominated by the “amyloid aggregation” concept where the oligomerization and accumulation of Aβ aggregates or fibrils in the brain ultimately lead to neuronal injury and death.

In AD patients, Aβ synthesis increases and the amyloidogenic peptide undergoes a highly dynamic self-assembly and stochastic process into non-fibrillar aggregates (off-pathway mechanism) and amyloid fibrils (on-pathway mechanism), resulting in the formation of various intermediates with differences in size, structure, and morphology [143], as described in Table 1. Aβ is able to form various protein species differing in size, morphology, solubility, and toxicity, which have been associated with both on- and off-aggregation pathways.

**Table 1:** Various types of Aβ species formed during aggregation based on their size and morphology reported both in vitro and in vivo

Aβ species		Characteristics	Size	References
Monomers		a) Soluble amphipathic IDP b) Unstable structure which may populate a diverse set of conformational states as opposed to a single dominant folded conformation c) Generated from APP d) Potential to form α-helical and β-sheet conformations e) When glycated may accelerate aggregation f) Non-toxic	MW ~ 4–5 kDa	[144–146]
LMW oligomers	Dimers	a) Smallest Aβ aggregates b) Hydrophobic core c) Highly stable and soluble d) [Aβ]DiY: possibly formed by a covalent bond due to a phenolic coupling of Tyr residues that further aggregates into soluble individual fibrils (in vitro, in vivo) e) [Aβ]Q-K: possibly formed by the action of transglutaminase which catalyzes the formation of an isopeptide bond between Gln15 and Lys16 that further aggregates into amorphous aggregates (in vivo) f) Potential basic constituent unit of fibrils or oligomers g) In AD human brains are composed of multiple Aβ monomers with different lengths h) May accumulate intracellularly i) Possibly the most potent toxic Aβ species j) Soluble	MW ~ 8–10 kDa Diameter 3–4 nm	[146–152]
	Trimers	a) Most abundant species produced and secreted by primary neurons in vitro b) May be the earliest Aβ aggregates formed, even before dimers, since they exist from childhood and their levels gradually increase with age, but there is no significant correlation between trimers and plaque deposits c) Considered aggregation units of multiple Aβ oligomers as hexamers and dodecamers d) First step to form amylospheroids species e) Appears to be dependent on the levels of Aβ production in vivo f) Soluble g) Possibly the most potent toxic Aβ species	MW ~ 12–15 kDa	[146–148, 153–156]
	Aβ‐derived diffusible ligands (ADDLs)	a) The acronym “ADDLs” was selected to emphasize the soluble, non-fibrillar, and ligand-like nature of these small globular off-pathway Aβ assemblies b) Contain predominantly trimers to dodecamers, but also higher-order species c) Enhance the glycogen synthase kinase‐3β (GSK‐3β) activity leading to the aberrant phosphorylation of Tau d) Inhibit long-term potentiation (LTP) e) Toxic f) Soluble	Low MW ADDLs: Diameter 1.5–3.5 nm Height 1.1–1.6 nm High MW ADDLs: Diameter 5–11 nm Height 4.5–6.5 nm	[157–161]
MMW oligomers	Globulomers	a) Globular shape, but not compacted b) Hydrophobic C-termini are supposed to extend to the interior of a globular structure while the more hydrophilic N-termini are exposed to the outer surface c) Residues 31–34 are the most rigid d) Contain mixed parallel and antiparallel β-sheet structure e) Although have substantial β-sheet content, they do not form fibrils and thus may be considered off-pathway aggregates f) Inhibit spontaneous synaptic activity in AD patients g) Prepared in vitro by incubating Aβ with SDS or fatty acids h) Soluble i) Toxic	Preglobulomer: MW 16–20 kDa Diameter 1–2 nm Globulomer: MW 38–48 kDa Large globulomer: MW 48–64 kDa Diameter 4–6 nm	[162–166]
	Dodecamers	a) Also known as Aβ*56 b) Sometimes considered as globulomers and type 1 Aβ oligomer c) Spherical shape d) Assembled by trimers e) Appear to be a dimer of hexamers f) Negligible in children and adolescents, and then steadily rises after the age of 40 g) Non-fibrillar Aβ aggregates h) Mainly located on the plaque free tissue and halo, but not in the plaque core i) Soluble j) Toxic	MW ~ 56 kDa Diameter 4–5 nm	[153, 156, 166–169]
HMW oligomers	Amylospheroids	a) Spherical in shape b) Might be formed by trimers units c) Off-pathway oligomers with no fibril formation d) Reduced level of β-sheet structure e) Equimolar Aβ and Zn^2+^ concentrations lead to spherical oligomers f) Bind to neurons g) Proteasomal inhibition causes an increase of amylospheroids in excitatory neurons and changes the subcellular localization from axons to dendrites h) May cause toxicity by involving Tau kinase I/GSK‐3β at the early stage of neurodegeneration i) Lead to an overload of cytoplasmic Ca^2+^ j) May cause mitochondrial dysfunction k) Toxic	MW ~ 160–670 kDa Diameter 10–35 nm	[121, 155, 170–176]
	Amorphous aggregates	a) Off-pathway oligomers b) Low β-sheet content c) Zn^2+^ and Cu^2+^ may induce off-pathway amorphous aggregates d) Some Aβ binding molecules induce amorphous aggregation e) Generally insoluble f) Non-toxic	Length 20 nm to µm	[177–184]
Protofibrils	Annular protofibrils (APFs)	a) Spherical ring-like shape morphology b) Form β‐barrel structures in the lipid membrane environment c) May be derived from the circularization of non-fibrillar Aβ assemblies d) Off-pathway oligomers since they do not convert into mature amyloid fibrils e) Potential role as membrane-disrupting pores or ion channels inducing cell death f) Might induce cellular Ca^2+^ influx by forming channels or by activating cell surface receptors coupled to calcium influx g) Also observed with other amyloidogenic proteins, such as α‐synuclein, PrP, and IAPP h) Prepared in vitro with Arctic variant (E22G) of Aβ40 and wt Aβ40 i) Prepared in vitro from the prefibrillar oligomers of Aβ42 by exposing them to a hydrophobic‐hydrophilic interface, such as lipid membrane j) Present in astrocytes of Aβ40 in AD brains k) Toxic	Outer diameter 6–10 nm Inner diameter 1.5–2 nm	[185–192]
	Linear protofibrils (LPFs)	a) Also known as large soluble aggregates b) Short, thin, elongated, and sometimes curvilinear c) Lack higher-order structure and periodicity as present in mature fibrils d) Strongly bind CR and Th-T e) High β-sheet content f) On-pathway precursor of amyloid fibrils g) Can accumulate in glial cells, associated with inflammatory responses, and present in activated astrocytes in AD brains h) Can be released through microglia-derived micro-vesicles, possibly contributing to extracellular spread and neuroinflammation i) Soluble protofibrils may be the most toxic Aβ species	Soluble 75–500 kDa Diameter 6–10 nm Length < 400 nm	[192–196]
Fibrils		a) Strongly bind CR and Th-T b) Each protofilament is composed of repeating Aβ units perpendicular to the fiber axis generating a cross-β structure c) Very high β-sheet content d) Generally formed by parallel β-sheet arrangements e) Stabilized by intermolecular hydrogen bonds f) Highly stable and insoluble g) Polymorphic with a number of 2–6 protofilaments h) Protofilaments often twisted around each other to form supercoiled rope-like structures i) Dehydrated core j) Primary Aβ form in amyloid plaques k) Associated with synaptic dysfunction in AD patients l) Observed in the vicinity of disrupted neurites plaques, regions of decreased spine density, and areas of neuronal loss m) Can be dissolved in formic acid n) Aβ42 fibrils are formed more rapidly than Aβ40 fibrils	Protofilaments: Diameter 2–5 nm Fibrils: Diameter 7–13 nm Length dozens µm	[197, 198]
Plaques		a) Large extracellular Aβ deposits b) Insoluble c) Final state of the Aβ aggregation process in vivo d) Composed of amyloid fibrils and/or amorphous aggregates e) Not toxic f) Surrounded by dystrophic dendrites, axons, activated microglia and reactive astrocytes	Diameter dozens µm	[121, 199]

In the on-pathway process occurs the formation of small intermediate species which are low-molecular-weight (LMW) oligomers with high β-sheet content. These LMW fibrillar oligomers are soluble and highly toxic [200]. These soluble oligomers, whether newly formed during AD development or released by mature deposits, interfere directly with cell and membrane function, most likely due to the exposure of hydrophobic groups on the oligomer surface, as well as due to the small size of these oligomers, with high diffusion coefficients [201]. The aggregation process normally proceeds via a nucleation-dependent polymerization reaction, forming insoluble, non-toxic, and mature fibrils through intermediate fibrillar species, such as highly ordered midrange (MMW) and high-molecular-weight (HMW) oligomers or aggregates, protofilaments, and protofibrils. The Aβ on-pathway mechanism englobes primary nucleation (monomer fibrillation and elongation) and secondary nucleation (fibril fragmentation and elongation) [201]. Aβ oligomers generated through primary nucleation are designated as type 1 oligomers and exhibit binding affinity to the A11 antibody, whereas those originating from secondary nucleation are classified as type 2 oligomers and bind to the OC antibody [167]. A11 and OC antibodies are known to recognize other amyloid-forming epitopes independently of the amino acid sequence of the protein [202, 203] and are currently being tested as anti-amyloid immunotherapies [204, 205]. As described by Liu et al. [167], type 1 oligomers are produced independently of Aβ fibrils, lack structural characteristics of amyloid fibrils, emerge prior to the formation of amyloid plaques, and are not localized around the cores of amyloid plaques. Conversely, type 2 oligomers are catalyzed by Aβ fibrils, share fundamental structural traits of amyloid fibrils, manifest only after the appearance of amyloid plaques, and are confined to the immediate vicinity of amyloid plaque cores.

In parallel, in the alternative off-pathway process, a variety of factors including cell components, metals, natural compounds, and environmental factors promotes the formation of spherical or globular aggregates that contain a reduced β-sheet content and non-fibrillar nature. Amorphous aggregates, in particular, are non-toxic species but generally insoluble. Some Aβ binding molecules have been reported to induce the formation of off-pathway amorphous aggregates [177–182].

Table 1 shows a compilation of different amyloidogenic and non-amyloidogenic Aβ species formed along the aggregation process derived from the analysis of recombinant-derived or chemically synthesized Aβ peptides or from AD brain extracts [206–212].

The general “traffic rules” for aggregation in vitro are very difficult to deduce since the aggregation process strongly depends on environmental conditions. Studies carried out in vitro have shown that the extent of aggregation depends upon many factors including solvent hydrophobicity, ionic strength, pH, temperature, pressure, agitation, thawing, drying, protein concentration, chemical modification, chemical cross-linking, salts, metal ions, surfactants, and cross-seeding polymerization, among others [213]. Regarding Aβ, its inclination for aggregation in vitro escalates in tandem with the hydrophobicity of the solvent and is contingent upon the storage conditions of Aβ preceding the onset of assembly. For example, Aβ aggregation when diluted in PBS is faster when Aβ is dissolved initially in NaOH, HCl, HFIP (1,1,1,3,3,3-hexafluoro-2-propanol), or DMSO (dimethyl sulfoxide) [214–217]. The presence of surfactants also favors Aβ aggregation [218, 219]. Moreover, high ionic strength facilitates the aggregation process, and the introduction of salts into Aβ solutions serves as a means to instigate aggregation [220, 221]. pH represents another crucial factor in the aggregation mechanism. Aggregation rates appear to accelerate below neutral pH, whereas highly acidic or alkaline conditions delay or inhibit fibrillization. Additionally, there is evidence suggesting that oligomeric intermediates formed under varying pH conditions exhibit distinct morphologies and levels of neurotoxicity [222–224]. Aβ fibrillization is also dependent on the concentration of Aβ peptide and the presence of pre-aggregated peptide forms, often referred to as seeds [225, 226]. The increase in temperature leads to conformation-partial unfolding that yields faster rates of aggregation [222, 227, 228]. Usually, agitation or stirring shortens aggregation dramatically [229]. Metal ions (Cu^2+^ and Zn^2+^) [230, 231] and impurities in Aβ preparations may also favor aggregation or lead to the formation of distinct oligomer morphologies [180, 181]. Chemical modification pathways have been shown to increase the aggregation tendency of Aβ. Such examples include dimerization [232], isomerization of aspartic acid residues [233], phosphorylation of serine and tyrosine residues [234, 235], nitration of tyrosine residues [234], racemization [236], and glycation [237]. Oxidation [238] and hydrolysis [239] are known to create the opposite effect, i.e., to slow the Aβ fibrillization process. Chemical cross-linking also contributes to enhancing Aβ aggregation [240]. Protein co-aggregation, heterogeneous or cross-seeding polymerization has been observed between Aβ peptides and different amyloid proteins facilitating the formation of Aβ aggregates and/or amyloid fibrils [63, 188, 241–248]. However, cross-seeding polymerization between Aβ peptide and Tau protein seems to promote Tau aggregation [103, 104].

Although the fundamental kinetic principles governing the self-assembly of Aβ remain consistent between in vitro and in vivo settings, the kinetics of the amyloid lifecycle under in vivo conditions are thought to differ significantly from the well-characterized in vitro conditions. Notably, one of the primary distinctions arises from the continuous and unrestricted production of Aβ monomers in vivo. Additionally, other significant disparities include the complexities introduced by genetic variations of the Aβ peptide or its precursor, along with various risk factors. Factors such as the cellular and extracellular environment, which influence amyloid aggregation kinetics, as well as spatiotemporal variations in Aβ monomer production leading to local monomer concentration fluctuations, further contribute to the differences. The rates governing the extracellular amyloid formation process and its kinetics are contingent upon the Aβ sequence and the prevailing environmental conditions. For instance, the aggregation propensity is influenced by the charge and exposed hydrophobic surfaces of the monomer. The lag phase may be shortened by increased fragmentation propensity or by protein co-aggregation. A defining characteristic of Aβ is its intrinsic disorder nature with the presence of extensive unstructured regions exposing hydrophobic areas, which are sufficient to initiate self-assembly. Furthermore, in vivo conditions such as post-translational modifications of Aβ, small elevations in temperature, transient pH changes during metabolic and neuronal activities [249, 250], the presence of other proteins, metal ions, natural compounds, and surface composition and properties can exert an influence on the aggregation of the Aβ peptide.

### Alzheimer’s Disease–Approved Drugs and Amyloid-β-Based Therapies

The German neuropsychiatrist Alois Alzheimer identified Alzheimer’s disease (AD) in 1906 after discovering cognitive impairments in a patient who died due to progressive brain function loss. Today, AD is an increasing global health challenge, with 55 million people suffering from this neurodegenerative amyloidosis. The patient with AD experiences memory loss, language impairments, behavioral and psychological symptoms of dementia, ultimately leading to death. Despite the rise in the number of cases in recent years and the consequential socio-economic costs, there is currently no efficient therapy to counteract or decelerate the development of AD.

To date, only seven drugs have received the approval of the US Food and Drug Administration (FDA) (Table 2) [251], and they only provide symptomatic relief and temporarily improve cognitive function. *Tacrine*, the first drug approved by the FDA in 1993 for mild to severe AD, was discontinued in 2013 mainly due to hepatotoxicity complications [252, 253].

**Table 2:** Drugs approved by the FDA and EMA for the treatment of Alzheimer’s disease: regulatory agency and year of approval, drug class and mechanisms of action, indications, clinical benefits, and more common adverse effects

Drug	Regulatory agency and year of approval	Drug class and mechanism of action	Indications	Clinical benefits	More common side effects
*Donepezil*	FDA (1996) EMA (1997)	AChE inhibitor—prevents the hydrolysis of ACh in the brain (1)	mild to severe AD	Although *donepezil* cannot alter the progression of AD, temporarily it can alleviate some symptoms by improving cognition and behavior	Nausea, vomiting, loss of appetite, and increased frequency of bowel movements
*Rivastigmine*	FDA (2000) EMA (2009)	AChE inhibitor—prevents the hydrolysis of ACh and BCh in the brain	mild to moderate AD	*Rivastigmine* does not cure AD, but it may improve memory, awareness, and the ability to perform daily functions	Nausea, vomiting, loss of appetite, and increased frequency of bowel movements
*Galantamine*	FDA (2001)	AChE inhibitor—prevents the hydrolysis of ACh and stimulates nicotinic receptors to release more ACh in the brain	mild to moderate AD	*Galantamine* will not stop the progression of AD, but long-term treatment improves cognition deficits in patients with this dementia [254]	Headache, constipation, confusion, and dizziness
*Memantine*	EMA (2002) FDA (2003)	NMDA receptor antagonist—blocks the toxic effects associated with the excess of glutamate and regulates glutamate activation in the brain (2)	moderate to severe AD	*Memantine* slows AD progression indicating that early treatment may maximize clinical success. *Memantine* offers significant benefits over time, enabling patients to maintain their independence, reducing caregiver burden, and postponing institutionalization [255]	Nausea, vomiting, loss of appetite, muscle cramps, and increased frequency of bowel movements
*Donepezil* + *Memantine*	FDA (2014)	(1) + (2)	moderate to severe AD	*Memantine* and *donepezil* lead to improvements in moderate to severe AD. *Memantine* improves global cognition, functional communication, and some behavioral symptoms (agitation and aggression) *Donepezil* improves neuropsychiatric, cognitive, and global functions, reducing the caregiver burden [256]	Nausea, vomiting, loss of appetite, increased frequency of bowel movements, headache, constipation, confusion, and dizziness
*Aducanumab*	FDA (2021)	Anti-amyloid monoclonal antibody—specific for the clearance of Aβ deposition in the brain, under the form of soluble oligomers and insoluble fibrils	MCI or mild AD	Patients treated with *aducanumab* show a reduction in Aβ plaques resulting in a slower progression of AD [257]	More severe symptoms: ARIA-E (brain edema or sulcal effusion) and ARIA-H (microhemorrhages or hemosiderin deposition into brain parenchyma) Less severe symptoms: headache, confusion, delirium, altered mental status, disorientation, dizziness, vision abnormality, nausea, diarrhea, hypersensitivity, and immunogenicity
*Lecanemab*	FDA (2023)	Anti-amyloid monoclonal antibody—binds with high affinity to soluble Aβ protofibrils, which in turn reduces the amount of Aβ deposits in the brain	adults with MCI or mild AD with confirmed presence of Aβ deposits	In an 18-month clinical trial, *lecanemab* has shown to reduce cognitive decline by 27% and to reduce the buildup of Aβ in the brain [258]	ARIA effects are characterized as ARIA with brain edema or sulcal effusions (ARIA-E), and ARIA with hemosiderin deposition including microhemorrhage and superficial siderosis (ARIA-H) Symptoms associated with ARIA may include headache, confusion, visual changes, dizziness, nausea, and gait difficulty Other symptoms as hypersensitivity reactions (angioedema, bronchospasm, and anaphylaxis), and infusion-related reactions may also occur

*ARIA*, amyloid-related imaging abnormality

The remaining oldest approved drugs are focused on symptomatological treatment acting at two levels, as through agonism of the cholinergic system or as antagonists of the N-methyl-D-aspartate (NMDA) receptor. The rationale for the use of *donepezil*, *rivastigmine*, and *galantamine* was based on the cholinergic hypothesis. This assumption was the first attempt to explain the pathophysiology of AD from a molecular point of view in the mid-1970s [81, 82]. Briefly, AD results from a selective loss in cholinergic neurons with decreased acetylcholine (ACh) synthesis. ACh is a neurotransmitter available in the brain with a significant role in the neuromodulation of learning, memory, and cognitive functions. Treatments that increase the cholinergic levels in the brain by inhibiting the biological activity of acetylcholinesterase (AChE) would be expected to provide clinical benefits. Therefore, AChE inhibitors are used to limit the degradation of ACh and are able to increase the function of neural cells by increasing the concentration of ACh [259]. The rationale behind *memantine* was related to the glutamatergic hypothesis [260, 261]. Briefly, glutamate is the main excitatory neurotransmitter in the brain. The glutamatergic overstimulation or the excessive glutamate levels due to its poor reuptake may result in neuronal damage, a phenomenon that has been termed excitotoxicity. Such excitotoxicity ultimately leads to a neuronal overload of calcium ions (Ca^2+^) that has been implicated in several neurodegenerative disorders [262]. Glutamate stimulates some postsynaptic receptors, including the N-methyl-D-aspartate (NMDA) receptor, which has been particularly implicated in the memory loss processes of the pathogenesis of AD. Treatments that block the effects of glutamate would be expected to provide clinical benefits. Thus, an uncompetitive NMDA-receptor antagonist could be of therapeutic value in AD, which is the case of *memantine* [261]. However, these drugs still show low efficiency in treating AD progression. *Donepezil*, *rivastigmine*, and *galantamine* can only alleviate some symptoms by improving cognition and behavior but do not alter AD progression (Table 2). In turn, it has been shown that *memantine* slows down AD progression, enabling patients to maintain their independence, while reducing the caregiver’s burden (Table 2). Combined therapies such as *memantine*-*donepezil* can also lead to some improvements in moderate to severe AD, by enhancing global cognition and functional communication, while *donepezil* improves neuropsychiatric, cognitive, and global functions (Table 2).

Nonetheless, given the poor efficacy of these AChE inhibitors and NMDA receptor antagonists, new drugs based on the design of anti-amyloid monoclonal antibodies (mAbs) have been developed. The first disease-modifying therapies (DMTs) for AD supporting the amyloid hypothesis have been recently employed. *Aducanumab* and *lecanemab* are two mAb drugs used for the clearing of the Aβ burden. Patients have demonstrated enhanced clinical outcomes and improved neuroimaging, as well as statistically changed biomarker levels, indicating a potential role in slowing down disease progression in AD individuals. *Aducanumab* prescription led to a reduction of Aβ plaques and to slower AD progression [257], whereas *lecanemab* has been shown to reduce cognitive decline by 27% and to reduce the buildup of Aβ in the brain [263]. However, patients have also exhibited an elevated probability of side effects, such as the occurrence of amyloid-related imaging abnormalities (ARIA) and infusion reactions when submitted to mAb therapies [264]. In addition, recent clinical studies on mAbs have shown that a slowdown of cognitive decline was not observed in women or APOE4 carriers [265].

Other drugs have been approved by other regulatory agencies. *Huperzine A* was identified by scientists in China in the 1980s as a potent and selective inhibitor of AChE [266], which has a mechanism of action similar to *donepezil*, *rivastigmine*, and *galantamine*. This drug was approved in 1994, and many preclinical studies and clinical trials have shown the potential effect of *huperzine A* in treating mild to moderate AD. Although *huperzine A* seemed to have some beneficial effects on AD, due to poor methodological quality and small sample size, there is still insufficient evidence for clinical recommendation [267]. *Sodium oligomannate* was also approved in China (National Medical Products Administration (NMPA)) in 2019 for mild to moderate AD to improve cognition [268]. However, other regulatory agencies have not approved it due to skepticism surrounding the clinical data supporting its potential benefits. Therefore, it is still undergoing phase 4 clinical trials necessary for regulatory approval in the USA and Europe (ClinicalTrials.gov Identifier NCT05058040, NCT05181475). The mechanism of action of *sodium oligomannate* is still unclear, and several possibilities have been proposed, including Aβ inhibition and disassembly [269], mediation of inflammatory responses to amyloid plaques [270], and protein binding inside neurons [271], among others.

According to the 2024 AD drug development pipeline [272], 164 clinical trials (phases 1, 2, and 3) were undergoing in 2023 for assessing 127 drugs for the treatment of AD and MCI, where phase 3 had 32 agents in 48 trials, phase 2 had 81 agents in 90 trials, and phase 1 had 25 agents in 26 trials. Of the 164 current AD trials, 35 (21.3%) are new according to the last Index Date (January 1, 2023 [273]). At present, anti-Aβ based therapies are the third most common therapies in clinical trials for the treatment of AD involving 23 agents in total (18.1%) (Table 3), where 7 agents are from Phase 3, 10 agents from Phase 2, and 6 agents from Phase 1.

**Table 3:** Anti-amyloid drugs active in clinical trials phases 3, 2, and 1 of AD on January 1, 2024. Disease-modifying therapies were divided into biologics (e.g., monoclonal antibodies, vaccines) and small molecules (e.g., drugs typically taken orally and less than 500 g/mol). Adapted from reference [272]

Phase	Agent	Therapeutic purpose	Mechanism of action	ClinicalTrials.gov ID
3	Aducanumab	Disease-modifying biologic	Anti-amyloid monoclonal antibody directed at plaques and oligomers	NCT04241068 NCT05310071
3	Donanemab	Disease-modifying biologic	Anti-amyloid monoclonal antibody specific for pyroglutamate plaque amyloid	NCT04437511 NCT05026866 NCT05508789 NCT05738486
3	Gantenerumab	Disease-modifying biologic	Anti-amyloid monoclonal antibody	NCT01760005
3	Lecanemab	Disease-modifying biologic	Anti-amyloid monoclonal antibody directed at amyloid protofibrils and amyloid plaques	NCT01760005 NCT03887455 NCT04468659 NCT05269394
3	Remternetug	Disease-modifying biologic	Anti-amyloid monoclonal antibody	NCT05463731
3	Solanezumab	Disease-modifying biologic	Anti-amyloid monoclonal antibody	NCT01760005
3	Valiltramiprosate	Disease-modifying small molecule	Prodrug of tramiprostate	NCT04770220
2	ABBV-916	Disease-modifying biologic	Anti-amyloid antibody	NCT05291234
2	ACI-24.060	Disease-modifying biologic	Vaccine stimulates antibodies against Aβ protein	NCT05462106
2	ALZN002	Disease-modifying biologic	Autologous Aβ mutant peptide-pulsed dendritic cells	NCT05834296
2	APH-1105	Disease-modifying small molecule	α-secretase modulator (APP secretase modulator)	NCT03806478
2	Lecanemab	Disease-modifying biologic	Anti-amyloid monoclonal antibody directed at amyloid protofibrils and amyloid plaques	NCT01767311
2	MIB-626	Disease-modifying small molecule	Sirtuin-nicotinamide adenine dinucleotide stimulator to enhance α-secretase	NCT05040321
2	PRI-002	Disease-modifying small molecule	Interferes with oligomerization of Aβ42 to prevent formation and enhance reduction of Aβ oligomers	NCT06182085
2	Trontinemab	Disease-modifying biologic	Monoclonal antibody directed at plaques and oligomers; “brain-shuttle” gantenerumab	NCT04639050
2	Valiltramiprosate	Disease-modifying small molecule	Aggregation inhibitor	NCT04693520
2	Varoglutamstat	Disease-modifying small molecule	Glutaminyl cyclase (QC) enzyme inhibitor to reduce production of pyroglutamate Aβ	NCT03919162 NCT04498650
1	ALN-APP	Disease-modifying biologic	RNAi to decrease APP and downstream Aβ-related events	NCT05231785
1	ALZ-101	Disease-modifying biologic	Aβ-directed vaccine	NCT05328115
1	AV-1959	Disease-modifying biologic	Anti-amyloid vaccine	NCT05642429
1	BMS-984923	Disease-modifying small molecule	Silent allosteric modulator (SAM) of mGluR5	NCT05804383 NCT05817643
1	Remternetug	Disease-modifying biologic	Anti-amyloid monoclonal antibody	NCT04451408
1	SHR-1707	Disease-modifying biologic	Anti-amyloid monoclonal antibody	NCT06114745

Some drugs have been reported to reduce Aβ in clinical trials. However, most of these studies did not achieve a significant improvement in the cognitive and memory function of patients with AD [274]. Aβ deposition was discovered in cerebrovascular patients in the 1980s and, although anti-amyloid agents possess a high risk of failure at a clinical stage, Aβ is still regarded by the scientific community as one of the major leading causes of AD and a promising target for anti-AD drug development. According to the amyloid cascade hypothesis, aggregation, and further accumulation of Aβ cause dysfunction of neurons and cell death, leading to AD development.

There are several therapeutic strategies based on this assumption targeting directly or indirectly Aβ that have been used in clinical trials. The most common are [274, 275]:

1. Immunotherapy (active anti-amyloid immunotherapy based on vaccines containing appropriate antigens that will promote the formation of antibodies against Aβ of multiple specificities, and passive anti-amyloid immunotherapy based on exogenous antibodies that will bind Aβ fibrils and thus prevent aggregation)
2. Decreasing the production of APP (inhibitors and modulators of γ-secretase, and β-secretase inhibitors)
3. Inhibiting the cleavage of APP (activation of α-secretase)
4. Inhibiting the aggregation of Aβ
5. Decreasing neurotoxicity
6. Promoting the degradation and clearance of Aβ.

Future research should be based on initiating Aβ clearance at an early stage since therapy has to be started before the development of significant neuronal loss [276]. AD pathogenesis is exceptionally intricate, involving numerous targets and pathways. This complexity poses a substantial challenge in the development of therapeutic strategies that address the underlying causes of neurodegeneration.

The inadequacy of the “one-drug-one-target” approach to drug design, coupled with the multifaceted nature of AD and of other amyloidogenic disorders, has prompted research into an alternative drug design strategy known as “designed multiple ligands”, “hybrid molecules”, or “multitarget-directed ligands” (MTDLs). This emerging approach focuses on developing pleiotropic ligands capable of simultaneously interacting with at least two therapeutic targets, thereby facilitating a synergistic effect. The pursuit of MTDLs has been particularly motivated by the quest for more effective AD treatments, leading to the proposal of numerous structures based on this polypharmacology concept [277, 278]. Among the most promising analogs are those derived from molecular hybridization, wherein multiple pharmacophores are combined to mimic the activity of parent compounds while maintaining a degree of selectivity toward the targeted receptors. These hybrid structures can be generated through (1) the use of a linker that connects and anchors the biologically active moieties, (2) the fusion of active segments, or (3) the simple merging of functionalities known to engage the targets. The rational design of these prospective drugs often draws inspiration from well-known or approved medications such as *tacrine*, *donepezil*, *rivastigmine*, or *galantamine*, as well as various natural bioactive derivatives recognized as amyloid disruptors, including resveratrol or curcumin [279–286].

## Conclusions

In 1906, Alois Alzheimer described the Alzheimer’s disease (AD) after detecting cognitive impairments in a patient who died of progressive loss of brain function. Nowadays, as defined by the NIA-AA, AD is morphologically identified by the simultaneous presence of extracellular deposits and neuritic plaques of Aβ amyloid, along with intraneuronal neurofibrillary tangles of hyperphosphorylated Tau protein. AD leads to progressive dysfunction and neuronal death, resulting in gradual loss of cognition and memory, along with personality and behavioral changes, ultimately leading to complete brain failure and death. Two main forms of AD have been defined: early-onset or familial AD (FAD) and late-onset or sporadic AD (SAD). Cerebrospinal fluid, plasma, and blood biomarkers used in AD diagnosis include Aβ42 concentration, Aβ42/Aβ40 ratio, phosphorylated Tau, or phospho-Tau (P-Tau), total Tau (t-Tau), P-Tau ratio, neurofilaments, synaptic proteins, presence of inflammatory markers, and activated astrocytes. Amyloid-β peptides (Aβ40 and Aβ42) are intrinsically disordered proteins produced by proteolytic cleavage of the APP protein. APP is a ubiquitous single-pass transmembrane protein that under physiological conditions plays an essential role in neural growth and repair. Nonetheless, different forms of Aβ, from soluble dimers to oligomers, either synthetic or derived from AD brains, can cause synaptotoxic effects and neuronal death. Aβ amyloid plaques and abnormal neurites are classified according to their relative amount, distribution, and morphology in (I) diffuse/pre-amyloid plaques, (II) neuritic plaques (non-cored and dense core), and (III) compact/burnt-out plaques. Aβ aggregation in vitro depends on several factors such as protein concentration, chemical modification, cross-linking, cross-seeding polymerization, presence of salts, metal ions, surfactants, solvent hydrophobicity, ionic strength, pH, temperature, pressure, agitation, thawing, and drying, among others.

AD etiology is still under severe debate and controversy but oligomerization and brain accumulation of Aβ aggregates and fibrils, leading to neuronal injury and death, certainly play a primary role. In this on-pathway, low-molecular-weight oligomers (highly toxic and β-sheet rich) are formed. Various Aβ species have been reported both in vitro and in vivo, ranging from monomers and low molecular weight species (dimers, trimers, and Aβ‐derived diffusible ligands) to medium (globulomers and dodecamers) and high molecular weight oligomers (amylospheroids and amorphous aggregates), protofibrils (annular protofibrils and linear protofibrils), fibrils, and plaques.

No effective treatment can reverse the progression of AD. So far, only a few drugs have been approved by the US Food and Drug Administration (FDA), either for the clearing of Aβ plaques or working as cholinergic agonists or NMDA receptor antagonists, although without achieving a significant improvement in cognitive and memory functions. Several new therapeutic strategies targeting Aβ aggregation, either directly or indirectly, are under clinical trials. The most common are immunotherapies against Aβ species, decreasing APP production (secretase inhibitors and modulators), inhibiting APP cleavage, inhibiting Aβ aggregation, decreasing neurotoxicity, and promoting Aβ degradation and clearance. However, the large number of side effects, the failure of the “one-drug-one-target” design, and the multifunctional nature of AD are inspiring the scientific community to investigate the effectiveness of other drug design strategies such as “multiple designed ligands”, “hybrid molecules”, or “multitarget-directed ligands”, in order to tackle this highly debilitating and fatal form of human dementia.

## Data Availability

No datasets were generated or analysed during the current study.
